# Effects of Phase Separation Behavior on Morphology and Performance of Polycarbonate Membranes

**DOI:** 10.3390/membranes7020021

**Published:** 2017-04-05

**Authors:** Alamin Idris, Zakaria Man, Abdulhalim S. Maulud, Muhammad Saad Khan

**Affiliations:** Department of Chemical Engineering, Universiti Teknologi Petronas, Bandar Seri Iskandar 31620, Malaysia; zakaman@petronas.com.my (Z.M.); halims@petronas.com.my (A.S.M.); khansaaad@gmail.com (M.S.K.)

**Keywords:** binodal curve, cloud point, phase separation, membrane morphology, performance

## Abstract

The phase separation behavior of bisphenol-A-polycarbonate (PC), dissolved in *N*-methyl-2-pyrrolidone and dichloromethane solvents in coagulant water, was studied by the cloud point method. The respective cloud point data were determined by titration against water at room temperature and the characteristic binodal curves for the ternary systems were plotted. Further, the physical properties such as viscosity, refractive index, and density of the solution were measured. The critical polymer concentrations were determined from the viscosity measurements. PC/NMP and PC/DCM membranes were fabricated by the dry-wet phase inversion technique and characterized for their morphology, structure, and thermal stability using field emission scanning electron microscopy, Fourier transform infrared spectroscopy, and thermogravimetric analysis, respectively. The membranes’ performances were tested for their permeance to CO_2_, CH_4_, and N_2_ gases at 24 ± 0.5 °C with varying feed pressures from 2 to 10 bar. The PC/DCM membranes appeared to be asymmetric dense membrane types with appreciable thermal stability, whereas the PC/NMP membranes were observed to be asymmetric with porous structures exhibiting 4.18% and 9.17% decrease in the initial and maximum degradation temperatures, respectively. The ideal CO_2_/N_2_ and CO_2_/CH_4_ selectivities of the PC/NMP membrane decreased with the increase in feed pressures, while for the PC/DCM membrane, the average ideal CO_2_/N_2_ and CO_2_/CH_4_ selectivities were found to be 25.1 ± 0.8 and 21.1 ± 0.6, respectively. Therefore, the PC/DCM membranes with dense morphologies are appropriate for gas separation applications.

## 1. Introduction

The phase inversion or separation process is one of the common techniques used for the development of asymmetric polymer membranes [1,2]. Generally, phase inversion occurs if there is a change in stability of the polymer solution. This, consequently, minimizes the free energy of the mixture which causes the solution to separate into two phases. The change in the stability of the polymer solution is accomplished by temperature variation, solvent evaporation, or by mass exchange with nonsolvent/coagulant bath [3]. The mass exchange with nonsolvent bath is regarded as nonsolvent induced phase separation, which is the most popular technique used to develop porous asymmetric membranes, while the solvent evaporation method is used to develop dense asymmetric membranes. For the nonsolvent induced phase separation or immersion precipitation method, there are three components involved in the phase separation, thus a ternary phase diagram is the most useful tool to describe the thermodynamic behavior of such ternary systems (nonsolvent/solvent/polymer). The immersion precipitation method creates change in the polymer concentration, hence the stability of the solution changes, as a consequence phase change or phase inversion occurs [4]. The phase inversion involves separation of polymer-rich and polymer-poor phases, for which mass transfer takes place involving the interchange of solvent and nonsolvent, by diffusion and convection [3,5].

**Figure 1:**
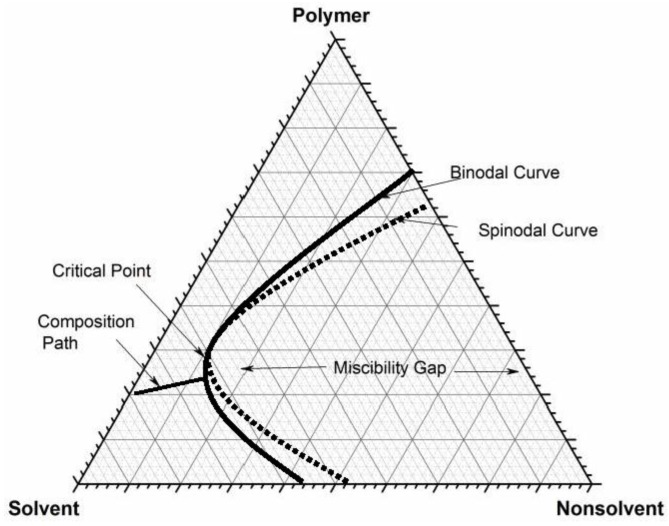
Theoretical composition path during phase inversion.

Strathmann et al. [2] were the first to explain the thermodynamic aspects of the fast and delayed demixing processes during membrane formation which lead to various types of membrane morphological structures [6]. The behaviors were described using a ternary phase diagram because of its convenience in investigating the thermodynamics of the membrane formation process, with a three component system i.e., nonsolvent/solvent/polymer. In the ternary phase diagram, the system is composed of a single-phase region and a two-phase region. In the single or one phase region, there exists miscibility of three components, while with the two phase region; the solution is separated into two phases, i.e., polymer-poor and rich phases. The boundary delimiting the liquid-liquid demixing is known as the binodal curve [4]. Within the binodal curve, the solution separates or demixes into two phases with different composition but in a state of thermodynamic equilibrium between each other for which their compositions can be shown by a tie line as in Figure 1. Several authors such as Wijmans et al. [7] and Klenin et al. [8] suggested two simple methods for determining the binodal curve by measuring the cloud points using rapid titration or turbidity measurement methods.

The cloud points represent the compositions at the binodal curve. The composition path of a polymer solution can be described in the ternary phase diagram after immersion in a nonsolvent bath. If the composition path is shorter, faster or instantaneous liquid-liquid demixing takes place. This requires less time to reach the binodal curve, so as to separate into two phases. If the composition path is longer, then there will be delayed liquid-liquid demixing. The differences in the liquid-liquid demixing rate produce different kinds of membrane morphology [9]. Several scholars observed various membrane structures based on the demixing rate or on the rate of polymer precipitation in a nonsolvent bath [10]. They have indicated that membranes with “sponge-like” morphologies were obtained with slow demixing rates. Since the demixing process is delayed, it requires a longer time for the formation of the membrane as the polymer precipitation is slow, therefore, a relatively dense top layer with sponge-like substructured membranes is obtained. On the other hand, with fast or instantaneous demixing rates, a finely porous substructure, with thin skin layered membranes is obtained. The formation of a porous substructure has been explained by several researchers; the macrovoids or pores substructures are governed by the precipitation or liquid-liquid demixing rate [2]. The initiation of such substructures is due to the surface tension gradient that causes hydrodynamic interfacial instabilities [11]. Further, the formation of such structures was associated with the intermolecular potential gradient near the interface [12]. Smolders et al. [13] related the formation of voids during the phase separation with the formation of nuclei at a stable composition for a longer period of time.

The binodal curve can be obtained experimentally by two methods, the turbidometric titration and cloud point method [14]. In the turbidometric titration, the procedure adds coagulant/precipitant incrementally to the dilute polymer solution and thus the intensity of the scattered light due to the turbidity can be measured and interpreted as a function of the amount of added nonsolvent/coagulant. However, for the cloud point method, a known quantity of polymer solution is put into a conical flask and drop wise addition of coagulant under continuous stirring can be carried out until the solution becomes cloudy or turbid. The stirring may continue for a few more hours to confirm whether the actual cloud point has been reached. From the weight of the added coagulant, the composition at the cloud point may be determined. Some authors however used coagulation values as a special case of cloud points for various solvent and nonsolvent pairs to indicate the level tolerance of the polymer solution, serving as indicators of the extent of the demixing rate that can possibly occur during phase inversion [15]. The coagulation or precipitation value is defined as the amount of coagulant in grams required to make 100 g polymer solution containing 2 g polymer become turbid [15,16]. Higher coagulation or precipitation values correspond to the larger coagulant tolerance of the casting solution which causes delayed demixing and lower values indicate a faster liquid-liquid demixing rate and hence low tolerance to coagulants.

The aim of this work is to study the effects of the phase separation behaviors on the morphology, thermal stability and performance of the polycarbonate membranes and thus to compare the applicability of the developed membranes for gas separation application. Two solvents dichloromethane and *N*-methyl-2-pyrrolidone were selected to form polycarbonate solutions with different phase separation behaviors. Their physical properties were measured to compute the solvent-polymer compatibility and determine the critical polymer concentration for membrane development. The phase behaviors of the polycarbonate solutions were studied for their liquid-liquid demixing rates or polymer precipitation by determining the phase boundary known as the binodal curve. The characteristic binodal curves for ternary water/solvent/polycarbonate systems were obtained experimentally by the cloud point method at room temperature. Moreover, two sets of membranes at around their critical polymer concentrations were developed by the dry-wet phase inversion method at customized experimental parameters. The morphology and structure of the membranes were characterized using Field Emission Scanning Electron Microscopy (FESEM) and Fourier Transform Infrared Spectroscopy (FTIR). The thermal stabilities of the membranes were analyzed using thermogravimetric analysis (TGA). Further, the gas separation performance of the membranes was evaluated at room temperature by measuring the permeance of N_2_, CH_4_, and CO_2_ gases using four channel membrane permeation cells.

## 2. Materials and Methods

### 2.1. Materials

Bisphenol A Polycarbonate (PC) (*M_w_* 64,000 g/mol, density 1.2 g/cm^3^), was purchased from LG-Dow Polycarbonate Ltd. (Seoul, Korea) *N*-Methyl-2-pyrrolidone (NMP) and Dichloromethane (DCM) were obtained from Merck Millipore (Selangor, Malaysia) and used as received without further purification, and double distilled water was utilized as a nonsolvent or coagulant. 

### 2.2. Preparation of Polymer Solution

PC polymer pellets were first dried in an oven at 90 °C for 24 h to remove moisture. Then, a known amount of dried PC pellets were dissolved in DCM and NMP solvents separately. The solutions were prepared on a weight basis with various polymer concentrations (wt %), in sealed glass bottles. The solutions were stirred for 24 h using a hot plate magnetic stirrer to achieve homogeneous and clear polymer solutions. All the dissolution and further experiments were conducted at a room temperature of 24 ± 0.5 °C.

### 2.3. Viscosity and Density Determination

The viscosity of the dope solution was determined using a rolling-ball viscometer (Model: Lovis 2000 M/ME, Anton Paar, Ashland, AL, USA). An amount of 100 μL sample solution was taken and put in a capillary tube with a metal ball and the sample viscosity was determined by considering the time taken for the ball to travel through the sample solution in the capillary tube.

The density of the dope solution was determined using a digital density meter (Model: DMA 5000 M, Anton Paar) with an oscillating capillary U-tube. The measurement of density is based on the frequency of oscillation. An amount of 1 mL sample solution is filled into a U-shaped oscillating capillary tube. The density meter is set in connection with the rolling ball viscometer. For the determination of viscosity and density, PC/DCM dope solutions with 3, 6, 9, 12, 15, 17, 19 and 21 wt % polymer concentrations and PC/NMP solutions with 3, 6, 9, 12, 15, 18 wt % polymer concentrations were used. The same sample solutions were also analyzed for the refractive index using a refractometer (RX5000α, Atago, Washington, DC, USA). The refractometer was first calibrated with distilled water of known refractive index. About 2–3 mL of the sample solution was drawn and put in the sample cone of the refractometer.

### 2.4. Cloud Point Determination

The cloud point data were determined by the titration method [17,18], where the coagulant water was added drop wise into the polymer solution under continuous stirring using an adjustable volume micropipette with 5 μL accuracy (Mechanical Micropipette, Favorit, Jakarta, Indonesia). The solution temperature was maintained at 25 °C with a water bath. The titration of the polymer solution against distilled water was stopped when the transparent solution turned visually cloudy or turbid. The cloudy solution was stirred for 30 min to check if the turbid solution reversed to a clear solution, for which additional drops of nonsolvent water were required to reach the true cloud point, but if the turbidity of the solution persisted then the composition of the mixture was considered as a true cloud point. For the determination of cloud point, PC/DCM dope solutions with 3, 6, 9, 12, 15, 17, 19 wt % polymer concentrations and PC/NMP solutions with 3, 6, 9, 12, 15, 18 wt % polymer concentrations were used.

### 2.5. Preparation of PC Membranes

The PC membranes were fabricated by the dry-wet phase inversion method, where PC solutions were first prepared by weighing a known amount of oven dried PC pellets, and dissolving in DCM and NMP separately in sealed bottles by mixing with a magnetic stirrer for 24 h at room temperature. The PC solutions were put in an ultrasonicator for 3 h to remove any possible bubbles created during the mixing process. The PC solutions were then cast on a glass plate using a doctor’s knife with a gap of 300 μm. Nitrogen gas was blown over the casted solution for a period of 15 s with immediate immersion into a water coagulation bath. The cast membranes were immediately immersed in a coagulation bath for 12 h to ensure complete mass exchange between the solvent and nonsolvent. Finally, the membranes were dried in a vacuum oven (i.e., 500 mbar vacuum and 120 °C) overnight to remove any residual solvents in the membrane matrix. Similar drying and pretreatment procedures are commonly reported in the fabrication of membranes [19,20].

### 2.6. Characterization

The developed PC membranes were characterized using VP-FESEM (Model SUPRA 55VPCarl Zeiss AG, Oberkochen, Germany) for their morphology and microstructure, where the PC membranes were first fractured with liquid nitrogen to obtain a smoother cross section. FTIR (Model Spectrum One/BX, PerkinElmer Inc., Waltham, MA, USA) was used to characterize the chemical structure and the changes induced due to the different types of solvents used during the preparation of the membranes. A thermogravimetric analyzer (TGA, STA6000, PerkinElmer Inc.) was utilized to study the thermal stability of the developed membranes, where 18 mg of the membrane samples were heated at a rate of 10 °C/min using nitrogen as carrier gas.

### 2.7. Performance Tests

**Figure 2:**
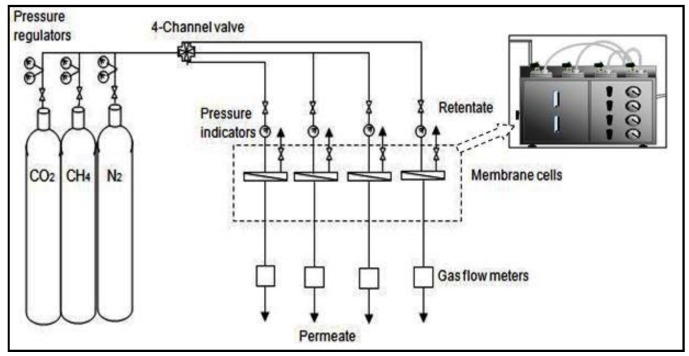
Four channel permeation cell set up.

The membrane performance tests were conducted in four channel permeation cells shown schematically in Figure 2. The membranes were first cut into 5.8 cm diameter pieces and assembled with the permeation cells. During the permeation test, the permeate flow rates were measured using a digital bubble flow meter. The gas permeance for the given membrane was then calculated as the permeate flow rate at STP per the effective membrane area per pressure difference. The ideal selectivity can be determined as the ratio of the permeances of the more permeating gas to the less permeating gas.

### 2.8. Calculation of Solubility and Interaction Parameters

#### 2.8.1. The Hansen’s Solubility Parameter

Hansen’s solubility parameters for the pure solvents and nonsolvent are obtained from the literature and that of the polymer is estimated from group contribution [21,22,23], where each structural group of the polymer is assigned to certain characteristic values and the corresponding dispersive, polar solubility, and solubility due to hydrogen bonding are calculated. Thus, the Hildebrand or the total solubility parameter is obtained by the expression:

(1) $$\delta =\sqrt{\delta_{d}^{2}+\delta_{p}^{2}+\delta_{h}^{2}}$$

The compatibilities among the components in ternary systems of coagulant/solvent/polymer, are mainly contributed due to their polar and non-polar interactions. The difference in Hansen’s solubility parameter serving as a measure of compatibility of components can be calculated using the expression:

(2) $$\Delta \delta_{j-p}=\sqrt{{\left(\delta_{d,j}-\delta_{d,p}\right)}^{2}+{\left(\delta_{p,j}-\delta_{p,p}\right)}^{2}+{\left(\delta_{h,j}-\delta_{h,p}\right)}^{2}}$$

where the suffix *j* and *p* correspond to the solvent or coagulant and polymer, respectively. Skaarup et al. [24] developed a solubility parameter distance, *R_a_*, between polymer and solvent, which serves as a measure of their compatibility or affinities according to their components’ Hansen solubility parameters. The solubility distance *R_a_* can be obtained by the following equation.

(3) $$R_{a}=\sqrt{4{\left(\delta_{d,j}-\delta_{d,p}\right)}^{2}+{\left(\delta_{p,j}-\delta_{p,p}\right)}^{2}+{\left(\delta_{h,j}-\delta_{h,p}\right)}^{2}}$$

Moreover, the compatibility or quality of the solvent with the given polymer may also be determined quantitatively by a convenient single parameter often known as the relative energy difference (*RED*) number [25,26], which is defined as the ratio of solubility parameter distance as given by Equation (3) to the interaction radius of the solute polymer.

(4) $$RED=\frac{R_{a}}{R_{o}}$$

where *R_a_* is the distance from solubility sphere, i.e., Hansen solubility parameter distance, while *R_o_* is the interaction radius of the polymer. Hansen reported the interaction radius for various polymers, for polycarbonate polymer the *R_o_* value was reported as 12.10 [27]. Thus, this value *R_o_* can be conveniently used in the comparison of the solvents. Based on the value of *RED*, one can determine whether the solvent is compatible or not. Generally, if *RED* is greater than 1 the material is non-solvent, or poor solvent, if *RED* is less than 1 and approaching 0, then it is regarded as a good solvent.

#### 2.8.2. The Solvent/Polymer and Nonsolvent/Polymer Interaction Parameters

According to the regular solution theory, the Flory-Huggins interaction parameters can be estimated from the solubility parameters. The simplest relationship between the solubility parameter and interaction parameters is given by [23]:

(5) $$\chi_{i3}=\frac{v_{1}}{RT}{\left(\delta_{1}-\delta_{2}\right)}^{2}\;i=1,\;2$$

where *χ_i_*_3_ is the Flory-Huggins interaction parameter, *v*_1_ is the pure molar volume for component 1, and *δ_i_* is the solubility parameter for component *i*.

Further, Hansen suggested a modified expression by incorporating a correction factor ‘α’ of unity in the given equation to estimate the Flory-Huggins interaction parameter from the Hansen solubility parameter [27]:

(6) $$\chi_{i3}=\alpha \frac{V_{1}}{RT}\left[{\left(\delta_{1,d}-\delta_{2,d}\right)}^{2}+0.25{\left(\delta_{1,p}-\delta_{2,p}\right)}^{2}+0.25{\left(\delta_{1,h}-\delta_{2,h}\right)}^{2}\right]\;i=1,\;2$$

The above two relationships are commonly utilized to estimate the Flory-Huggins interaction parameters for nonsolvent/polymer and solvent/polymer interaction parameters. Moreover, Wei Y.-M et al. [28] reported that the suggested equation by Hansen was in good agreement with the experimental results for ternary systems. Thus, in this work, Equation (6) was considered to estimate the Flory-Huggins interaction parameters.

Moreover, the solvent-polymer interaction parameter, *χ*_23_, also gives quantitative information about the degree of interaction of a polymer-solvent system. If *χ*_23_ is less than 0.5, the solvent is regarded as good for the polymer, and if it is higher than 0.5, it is poor solvent. Therefore, the *χ*_23_ may conveniently be used to determine the choice of solvent for a given polymer.

## 3. Results and Discussion

### 3.1. Solubility Behavior of PC

In order to determine the relative compatibility of PC in DCM and NMP as well as the nonsolvent water, Hansen’s solubility parameters were used to calculate the interaction parameter *χ*_23_, solubility distance *R_a_*, solubility difference *∆δ_j–p_*, and the *RED* values for the solvents DCM and NMP and nonsolvent water, which are presented in Table 1. The differences in their respective solubility parameters are obtained from Hansen’s solubility parameter according to Equation (2). While the *RED* values are obtained as the ratio of the Hansen’s solubility parameter distance to the interaction radius of the polymer as described by Equation (4) and the Flory-Huggins or solvent-polymer *χ*_23_ and nonsolvent-polymer *χ*_13_ interaction parameters were calculated using Equation (6). The solubility differences of PC and the solvents revealed that the interaction of PC with DCM is very efficient and almost 2.84 times more compatible than with NMP solvent. These results are in good agreement with the polarity judgment between polymer and solvents and Table 1 shows that the polarity differences of PC and DCM are narrow compared to NMP. It can be observed that low polarity differences between PC and solvent led to lower solubility differences. This generalization was made based on the less the difference in their respective solubility parameters, the better is the compatibility of the polymer-solvent pair. For which the DCM solvent was observed to be a good solvent for the dissolution of PC polymer. This observation can also be supported by the theoretical value of the interaction parameter (*χ*_23_ = 0.23 <0.5) which is regarded as a good solvent for PC polymer. However, the *χ*_23_ value of NMP is higher than 0.5 which is categorized as a poorer solvent. The results revealed that the solubility distance, *R_a_*, of DCM lies within the interaction region of PC while for NMP *R_a_* is closer to the boundary of the interaction region according to the Hansen solubility plot. Therefore, it can be conveniently concluded that NMP is a poorer solvent compared to DCM solvent for the dissolution of PC polymer. Moreover, the relative energy difference (*RED*) is a quantitative method to determine whether a given material is a solvent or nonsolvent for the given polymer. If RED is less than unity and approaching zero, then the material is a good solvent, and if it is approaching unity, then the solvent is poorer, otherwise the material is nonsolvent. Thus, based on the results of NMP the *RED* value is 0.76 which is approaching unity, and for DCM the *RED* value is 0.27 which is less than unity, this confirms that both materials are solvents. However, the *RED* value for water is 3.15 which indicates that water is highly nonsolvent or a coagulant for PC.

### 3.2. Cloud Points Binodal Curve

**Figure 3:**
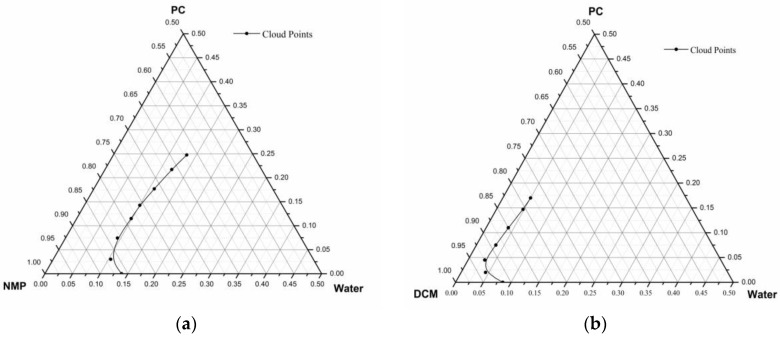
Cloud point data (**a**) water/NMP/PC and (**b**) water/DCM/PC.

Cloud points for the ternary systems water/NMP/PC and water/DCM/PC were experimentally determined at room temperature. The results are plotted in a ternary phase diagram shown in Figure 3a,b, respectively. These cloud points represent the binodal liquid-liquid demixing boundary of the system that exists in thermodynamic equilibrium during phase separation processes of membrane formation. Comparing the phase diagram of water/NMP/PC and water/DCM/PC, with a change in solvent type, the binodal curve shifts towards the polymer/solvent axis. This shift also changes the composition path for a given polymer-solvent pair to reach the state of thermodynamic equilibrium which takes place at the binodal curve. As for the case of the PC-DCM pair, the composition path is shorter compared to the PC-NMP pairs. The results shown in Figure 3 revealed that the PC-DCM pair demixing rate is remarkably faster, however the PC-NMP pair demixing rate is observed to be slower than the PC-DCM pair. On the other hand, the miscibility gap of the PC-DCM pair is large compared to the PC-NMP. The differences in terms of compositional path, demixing rate and miscibility gap can readily be observed in Figure 3a,b. It indicates the level of nonsolvent (water) tolerance, which is used to characterize the liquid-liquid demixing rates for the pair of solvent and nonsolvent during the phase inversion process. It can be concluded that for water as the strongest coagulant, DCM shows a faster demixing rate compared to NMP solvent. These results can be further justified by their difference in the interaction parameter *χ*_23_ shared between the solvent and polymer.

It can be concluded that for different pairs of polymer-solvent, the phase separation behavior or the precipitation pattern exhibited were found to be different. The variation observed depends on several factors including the interaction parameters and the solubility parameter differences.

**Figure 4:**
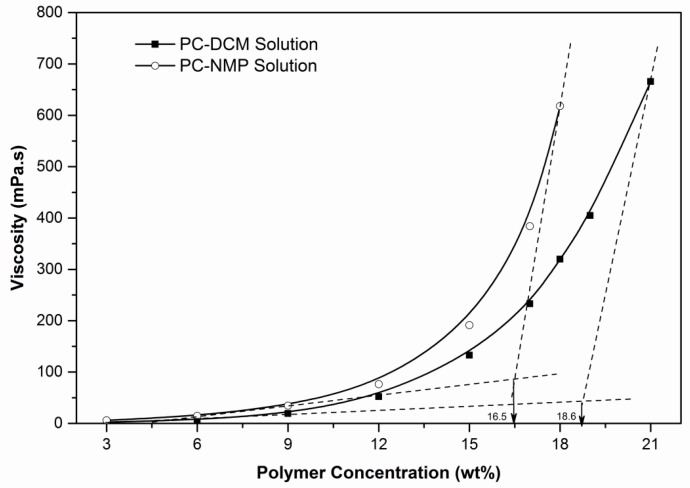
Critical polymer concentrations for PC-NMP and PC-DCM solutions.

The phase separation behavior of the polymer solution plays an important role in determining the morphological structure of membranes fabricated by nonsolvent induced phase separation i.e., the immersion precipitation method. Further, the solution properties such as intrinsic viscosities, refractive index, and density affect the interaction parameters which in turn influence the phase separation behavior [29]. Most importantly, during the preparation of asymmetric membranes, determination of appropriate polymer concentrations becomes important prior to the development of the membrane. Therefore, the critical polymer concentrations at which the membrane can be prepared were determined experimentally by measuring the intrinsic viscosities of polycarbonate solution prepared at different concentrations [30]. Two solvents DCM and NMP were compared and their critical polymer concentrations were determined by plotting the viscosities against polymer concentrations, and then the critical polymer composition can be obtained at the intersection point of the two tangents on the viscosity curves, as shown in Figure 4. Higher polymer concentration solutions beyond the critical viscosity are usually difficult to obtain and so fabricate the membrane at room temperature In addition to this; the flexibility of the resulting membrane would be more rigid affecting the gas permeation behavior of the membranes. However, at a polymer concentration lower than the critical viscosity, the membrane results in having reduced gas diffusivity, in addition to weaker mechanical strength [31]. Thus, according to the viscosity results, the critical polymer compositions for PC-DCM and PC-NMP were found to be 18.6 and 16.5 wt %, respectively. Therefore, it can be recommended that membranes for gas separation application may be prepared at ±1 wt % of the critical polymer concentration. Table 2 shows the viscosity, density, and refractive indexes of the PC-NMP and PC-DCM solutions. On comparison of the physical properties of the solutions, the densities of the PC-DCM solutions are higher than PC-NMP solutions contributed by the differences in the individual solvent densities. Further, the difference of densities between PC-DCM and PC-NMP solutions is probably attributed due to the polarity similarity of PC-NMP compared to the PC-DCM as the polarity change between PC-DCM is lower than for PC-NMP solution. This justification is also in good agreement with the concept of the miscibility gap observed in the phase diagram, which led to a larger miscibility gap in the ternary system of PC-DCM compared to PC-NMP solution. Moreover, the refractive indexes of PC-DCM solutions are lower which may yield to high transparency PC membranes compared to those of PC-NMP solutions refractive indexes. The results of RI are in good agreement with PC-DCM solubility compatibility; further the viscosity results support this agreement with increased viscosity of PC-NMP at identical polymer concentration compared to the viscosity of PC-DCM solutions. Thus, the critical viscosity of PC-DCM is estimated graphically to be higher than PC-NMP solutions.

The difference in viscosity between PC-DCM and PC-NMP could be related to the dispersion of the PC chains, which may be associated with the twisting positions of the methyl and bisphenol groups, and perhaps the dispersion of the polymeric chain is more aggressive in DCM solvent. Thus the viscosity of PC-DCM is lower than PC-NMP solution.

### 3.3. FESEM Analysis

**Figure 5:**
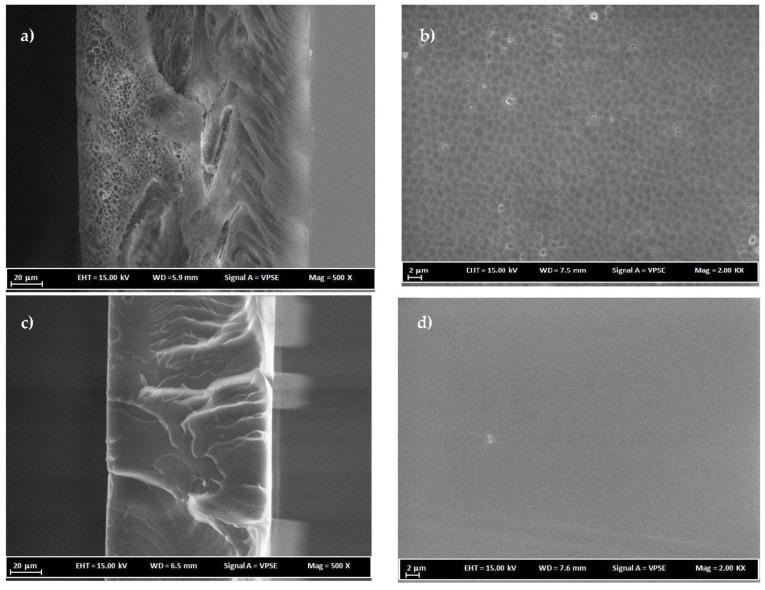
FESEM cross-sectional and surface images of membranes (**a**,**b**) 16 wt % PC/NMP and (**c**,**d**) 16 wt % PC/DCM.

PC membranes prepared at around the critical polymer concentrations with NMP and DCM solvents were analyzed for their morphological characterization using FESEM. Figure 5 shows the cross-section and surface FESEM images for 16 wt % PC/NMP and PC/DCM membranes. It can be clearly observed that the PC/NMP membrane possesses a sponge-like porous substructure beneath the denser top layer. On the glass side of the membrane macrovoid structures can be observed. However, for the 16 wt % PC/DCM membrane, the morphology appears to be an excessively dense top layer and dense substructure. The reason behind such morphological changes of PC membranes is attributed to the difference in phase separation behavior induced by different types of solvents. The system of water/NMP/PC exhibited longer composition path with delayed liquid-liquid demixing, resulted in sponge-like porous and macrovoid substructures on membrane morphology. However, with the ternary system water/DCM/PC, an instantaneous or fast demixing rate was observed, as a result, denser membrane morphologies were obtained. Similar structures were reported by Smolders et al. [13].

**Figure 6:**
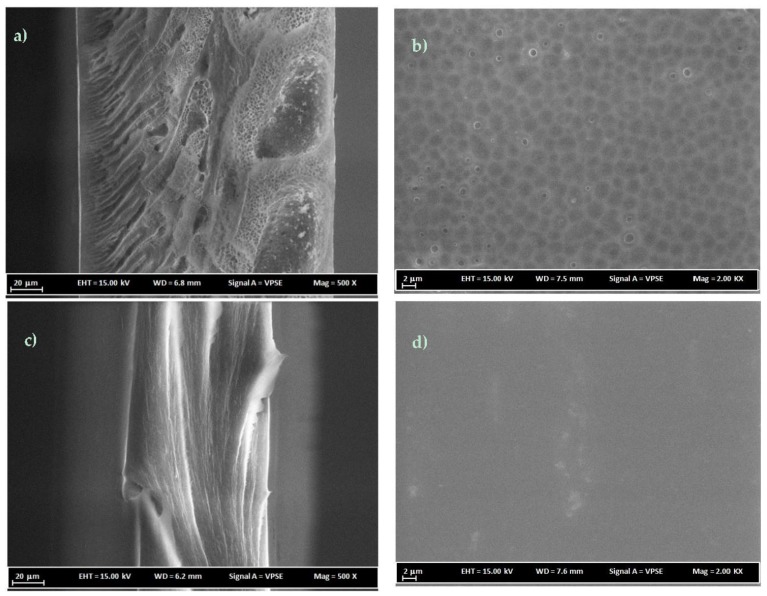
FESEM cross-sectional and surface images of membranes (**a**,**b**) 18 wt % PC/NMP and (**c**,**d**) 18 wt % PC/DCM.

Figure 6 shows FESEM images for membranes prepared at 18 wt % PC polymer concentrations. PC/DCM membranes as shown in Figure 6c,d appear to have denser membranes. On the structure of membrane morphology, the direction of casting is manifested as dense layers with the membrane matrix due to instantaneous or fast evaporation of DCM solvent. Therefore, the formation of dense PC/DCM membranes is contributed to by the solvent evaporation step involved in the procedure and by liquid-liquid demixing i.e., solvent–nonsolvent exchange. During the evaporation step, nitrogen gas was blown over the casted solution for a fraction of time i.e., 15 s, which evaporated an appreciable amount of the volatile DCM solvent before immersion into the nonsolvent coagulation bath.

**Figure 7:**
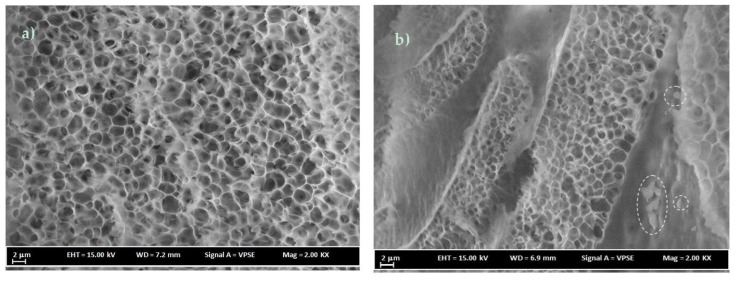
FESEM cross-sectional images of membranes (**a**) 16 wt % PC/NMP and (**b**) 18 wt % PC/NMP, the traces of residual solvent are shown by the circled area.

A PC/NMP membrane with 18 wt% polymer concentration is shown in Figure 6a,b, the difference in the morphology of the membrane prepared at different polymer concentrations as shown in Figure 5a,b appears to be significant, leading to intensive and smaller dimensions of the sponge-like porous and macrovoids substructures in the case of PC/NMP membranes. Viewing the surface images of Figure 5b, Figure 6b, the increase of polymer concentrations resulted in increasing the pore size of the microvoid substructures. The differences are more visible at high magnification images as shown in Figure 7 where the sponge-like pores and macrovoids can be easily seen and compared. Moreover, traces of residual solvents can be observed as indicated by the drawn circles in Figure 7b. The spaces between the layers of sponge-like porous substructures are the macrovoids. 

### 3.4. FTIR Analysis

**Figure 8:**
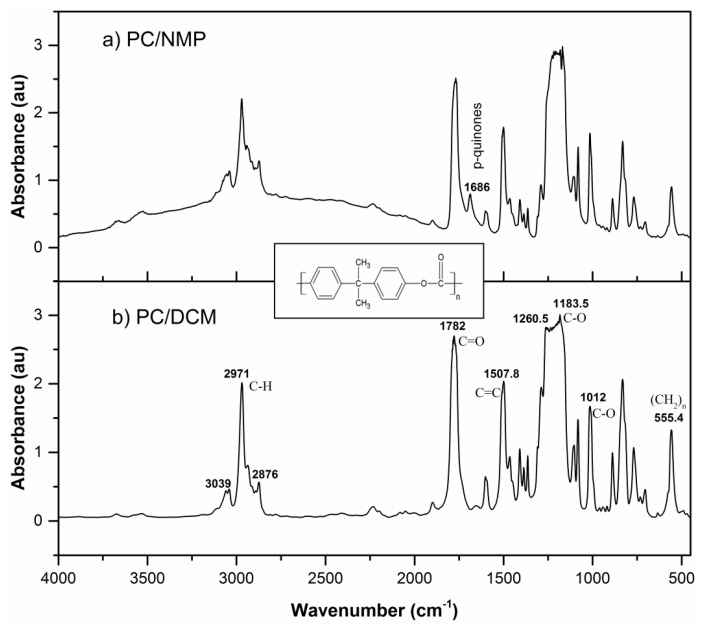
FTIR Absorption spectra for PC membranes prepared by (**a**) 18 wt % PC/NMP and **(b**) 18 wt % PC/DCM.

Figure 8 shows the FTIR absorption spectra for PC membranes prepared using DCM and NMP as solvent. All the characteristic absorption peaks were identified in both the spectra and their respective functional groups assigned. The characteristic peaks of the range of frequencies 3200–2800 cm^−1^ is assigned to C–H stretching which is identified at 3039, 2971, and 2876 cm^−1^. The characteristic bands in the range 1820–1680 cm^−1^ are assigned to the carbonyl C=O stretching detected peak at 1782 cm^−1^. The band at 1507 cm^−1^ is assigned to C=C stretching. The intensive characteristic absorption bands in the range 1300–1000 cm^−1^ are assigned to C-O stretching bonds, while the peak at 1260.5 cm^−1^ belongs to C(=O)–O stretching and at 1183.5 cm^−1^ to O–C=C asymmetric stretching. The absorption band at 1012 cm^−1^ is assigned to C–O while the band at 555.4 cm^−1^ belongs to (CH_2_)*_n_* functional groups with n > 4 [32].

Comparing the FTIR absorption spectra for both the membranes, a new absorption peak at 1686 cm^−1^ can be observed with the PC/NMP membrane, this peak is identified as para-quinones which have formed as a result of the interaction of the residual solvent with the aromatic phenol rings [33,34]. Moreover, the shift in the absorbance of the PC/NMP membrane indicates that there is presence of traces of residual solvent in the membrane matrix.

### 3.5. Thermal Analysis

The thermal stability of the PC/NMP and PC/DCM membranes, i.e., degradation temperatures were investigated using TGA analysis. Further, the possible presence of residual solvent in the polymer matrix was also studied using TGA curves. This is because the residual solvents can potentially interact with the polymer matrix which may further lead to a negative contribution to the overall performance and strength of the membranes. It affects the permeation behavior by blocking the sorption site and decreasing the free volume of the polymer membrane matrix [35].

**Figure 9:**
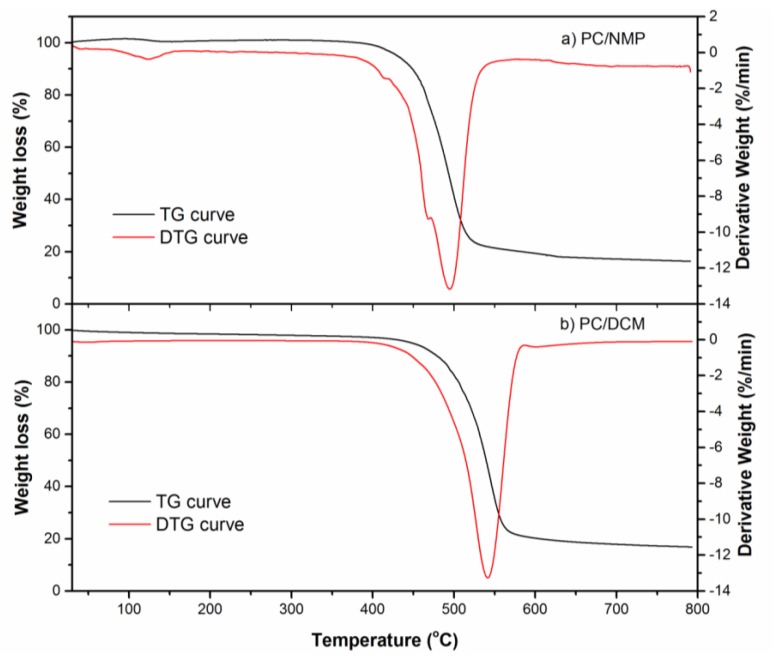
TGA and DTG curves for PC membranes prepared by (**a**) 18 wt % PC/NMP and (**b**) 18 wt % PC/DCM.

The TGA curves of the PC membranes prepared using NMP solvent along with their derivative curves are shown in Figure 9 for the temperature range of 30–800 °C. It can be readily observed that thermal behaviors of the PC membranes prepared by NMP and DCM solvents have remarkable variations. Starting with moisture evaporation step, i.e., at 108 °C, the PC/NMP membrane exhibited a very slight weight gain, which is probably due to the interaction of residual solvent in the membrane with the polymer chains of the membrane. This phenomenon resulted in the continuous swelling of the membrane matrix, however such maximum weight gain was observed at maximum at 93.72 °C, with 1.5011% gain. Beyond which, the continuous weight lost can be observed clearly by the negative derivative on the DTG curve at temperature 125.06 °C. However, for the case with PC/DCM membrane, the TG curve indicates that there is no residual solvent in the membrane matrix.

For the given polymer concentration (18 wt %) and coagulation time i.e., 12 h, the membrane prepared from the PC/DCM pair, demixed at a faster rate when it was brought in contact with the coagulant water, which led to complete removal by exchange of the solvent from the polymer. This was further justified with the mechanism of phase separation using the binodal curves described previously. The membrane prepared from PC/NMP dope solution, however, showed traces of residual solvent, which was confirmed structurally using FTIR results and visually observed in the FESEM images. The reason for the presence of residual solvents is that the NMP solvent was not removed completely from the PC polymer matrix during the course of the coagulation period of 12 h, which was caused by a delayed demixing rate. Further, this can be explained in terms of the miscibility gap and the compositional path differences in the ternary diagram with a shorter composition path and larger miscibility gap observed with the PC/DCM pair compared to that of the PC/NMP pair.

The presence of the residual solvents diminishes during the devolatilization step, thus its presence may not have a direct and profound impact on the thermal stability of the membranes. In fact, it is the morphological structure of the membrane that has a direct impact on the degradation temperatures. As shown in Table 3, the initial and maximum degradation temperatures of PC/NMP membrane has been reduced by 4.18% and 9.17%, respectively when compared with PC/DCM membrane. This can be further justified according to the FESEM images, membranes prepared using NMP solvent resulted in a porous substructure, while the membranes prepared with DCM yielded dense membranes. The porous membrane has a larger surface area to thermally decompose at a particular temperature, resulting in more weight loss. For instance, at a temperature of 472 °C, the amount of weight losses for PC/NMP and PC/DCM membranes are 26.26% and 8.52%, respectively. This indicates that the morphological structure of the membrane has a direct impact on the degradation temperatures and the final residue weights.

### 3.6. Performance Test

**Figure 10:**
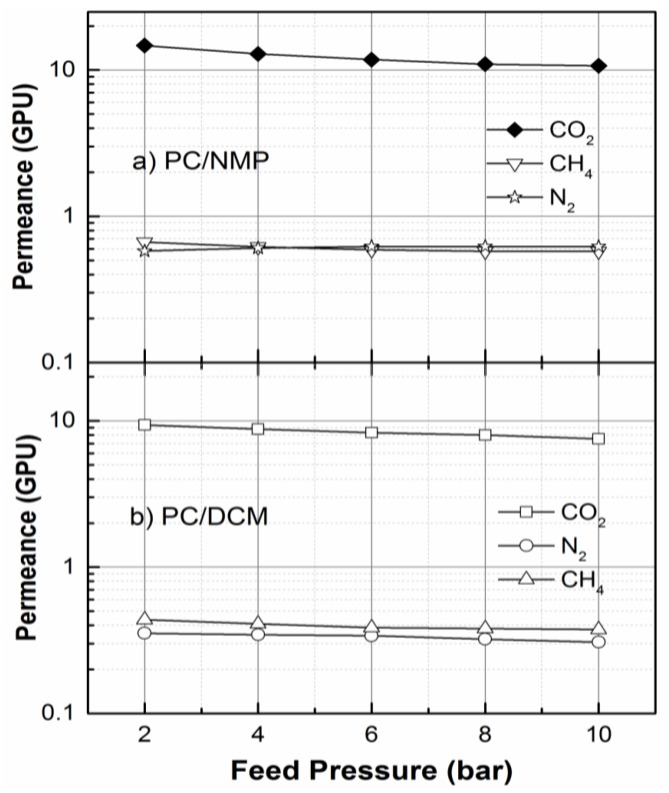
Gas permeances for (**a**) 18 wt % PC/NMP and (**b**) 18 wt % PC/DCM membranes at various feed pressures.

To investigate the effect of the morphological microstructure on the gas permeation performance, the PC/NMP and PC/DCM membranes were tested for their permeation behaviors to N_2_, CH_4_, and CO_2_ gases. Figure 10 shows the permeance of the PC membranes to N_2_, CH_4_, and CO_2_ gases at various feed pressure from 2 to 10 bars and at room temperature. On comparison of the gas permeances of the membranes, it can be observed that the PC/NMP membrane exhibited increased permeance to all gases as compared to that of PC/DCM membrane. The change in the permeance values is obviously attributed to the changes in the morphological microstructure of the membrane. As the PC/NMP membrane possesses pores, the resistance/barrier to the permeation of gases through the effective membrane thickness is reduced, which yields increased permeance. Moreover, it can be readily observed that the permeances of PC/NMP membrane decreased with the increase in feed pressures. However, in the case of PC/DCM membrane, the decrease in permeance to CO_2_ gas with feed pressures was quite significant. Such a decrease in permeance of CO_2_ at higher pressures is associated with the driving force applied which was greater than the resistance developed by the membrane against the penetrating gas. However, the N_2_ and CO_2_ gas permeation behaviors were consistent at various feed pressures.

According to the gas transport mechanism, PC/DCM membrane follows the solution-diffusion mechanism, where the gas is first absorbed, diffuses through the membrane thickness and finally gets desorbed on the permeate side of the membranes. For this mechanism the solubility and diffusivity of gas throughout the membrane thickness are the main factors of gas permeation. Solution-diffusion mechanism impose higher resistance to the penetrating gas, the gas transport is highly dependent on the solubility or interaction of the diffusing gas with the polymer membrane matrix. However, in the case of PC/NMP, the mechanism followed seems to be combined mechanisms i.e., solution diffusion on the top layer and Knudsen diffusion on the pore and microvoids or macrovoids substructures. Such mechanisms were briefly demonstrated for He and N_2_ gas permeation through dense and highly permeable or porous membranes by Albo et al. [36] For this reason, PC/DCM showed reduced permeances when compared with PC/NMP. The solution diffusion membranes usually exhibit low permeation rate and high selectivities while the Knudsen-diffusion membranes exhibit higher permeation rates and low selectivities [37].

The intrinsic properties of the penetrating gas such as condensability, polarity and kinetic diameter or molecular size have a direct impact on their permeation through the membrane matrix. Generally, since CO_2_ gas has a smaller molecular size, it is expected that it would permeate at faster rates compared to other gases such as CH_4_ and N_2_. For the dense PC/DCM membranes, the trend of permeance to the respective gases exhibited a direct proportionality relationship with the molecular sizes of the penetrating gas. However, with the sponge-like pores and macrovoid membrane morphologies, as observed in PC/NMP membrane shown in Figure 7, the role of the molecular size of the gas molecules is insignificant.

**Figure 11:**
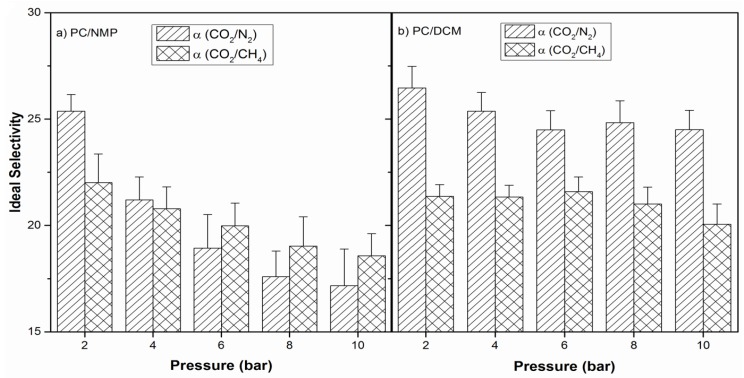
Ideal selectivity of PC membranes prepared (**a**) 18 wt % PC/NMP and (**b**) 18 wt % PC/DCM.

The calculated ideal selectivities at various feed pressures are shown in Figure 11 for both the PC/NMP and PC/DCM membranes. The tremendous decrease in ideal selectivity of PC/NMP membrane is mainly due to the presence of nonselective micro porous structures in the membrane matrix. With the increase in feed pressure, the change in permeance resulted in decreased ideal selectivities. This is because; the porous and macrovoids substructures in the membrane structures tend to collapse at high pressures affecting the overall performance of the membrane. However, with the PC/DCM dense membrane, the selectivities were observed to be somehow consistent with average ideal CO_2_/N_2_ and CO_2_/CH_4_ selectivities of 25.1 ± 0.8 and 21.1 + 0.6 respectively.

## 4. Conclusions

Polycarbonate solutions were prepared using NMP and DCM solvents, which exhibited different phase separation behaviors when nonsolvent water was added, or when the solution was immersed into coagulant water. Experimentally, demixing behaviors were studied by determining the cloud points as well as the physical properties such as intrinsic viscosities, refractive indexes, and densities of the polymer dope solutions. Theoretically the compatibility of the solvents with PC polymer was estimated by their solubility parameter difference, relative energy difference (*RED*), and the interaction parameters. The binodal curves were obtained from cloud point data measured by a titration method on the ternary water/solvent/polymer system at room temperature. The cloud points were determined for various polymer concentrations in NMP and DCM solvents, based on the results of cloud points, the characteristic ternary phase diagram i.e., the binodal curve were plotted for water/NMP/PC, and water/DCM/PC ternary systems. Results indicate that the PC-DCM pair exhibited a shorter compositional path on the binodal curve, while the PC-NMP pair were observed to have a longer composition path to reach the binodal curve. This indicates that PC-NMP solution has higher tolerance to the coagulant water, which in turn shows delayed demixing rate occurring between solvent and nonsolvent during the phase inversion process of membrane formation. However, PC-DCM indicates a fast demixing rate compared to PC-NMP with little tolerance to the nonsolvent water, thus forming the membrane in a short period of time. Further, experimentally PC-NMP and PC-DCM solutions were compared by measuring their intrinsic viscosities at room temperature. Using the intrinsic viscosities, the critical polymer concentration for the development of asymmetric polymeric membranes was determined graphically as 16.5 and 18.6 wt % for PC-NMP and PC-DCM pairs, respectively.

The PC/NMP and PC/DCM membranes at polymer concentration at around the critical polymer concentrations i.e., at 16 wt % and 18 wt % PC were prepared by the dry-wet phase inversion technique and characterized by their morphology, structure, and thermal stability using FESEM, FTIR, and TGA respectively. The morphological analysis indicates that the PC/NMP membrane is an asymmetric membrane with sponge-like porous and macrovoids substructure, while the PC/DCM membrane is observed to be a dense asymmetric PC membrane. Traces of residual NMP solvents were observed in the FESEM images for PC/NMP membranes. Further such residual solvents were also confirmed by FTIR analysis, showing new peaks as a result of the residual solvent interaction with the polymer chains. TGA analysis revealed a very slight weight gain as a consequence of residual solvent interaction with polymer membrane matrix at temperatures around 100 °C. Thus, PC/DCM membrane exhibited better thermal stability when compared with PC/NMP membrane. The gas separation performances of the membranes were evaluated by measuring the permeance of N_2_, CH_4_, and CO_2_ gases, through the membranes. In both the membranes, the order of permeance are observed as *P/l*(CO_2_) *> P/l*(CH_4_) > *P/l*(N_2_) at lower feed pressures below 4 bar. However, with the increase in feed pressure above 4 bar, the order of CH_4_ and N_2_ was reversed with PC/NMP membranes. Moreover, the CO_2_/N_2_ and CO_2_/CH_4_ ideal selectivities of PC/NMP membrane decreased significantly with the increase of feed pressures, while for the PC/DCM membranes the ideal selectivities were found to be slightly consistent throughout the feed pressure tested. Therefore, according to analysis of the membrane properties, and performance, the PC/DCM membrane was found to be an appropriate membrane for gas separation with better morphological compactness, thermal stability, and separation factor.

## Figures and Tables

**Table 1:** Solubility parameter of pure components [27] and calculated solubility parameters difference, Hansen’s solubility parameter distance, RED, and interaction parameters.

Material	PC	NMP	DCM	Water
*V_m_* (mol/cc)	211.67	96.50	63.9	18.02
*δ_d_* (MPa)^1/2^	17.95	18.00	18.2	15.50
*δ_p_* (MPa)^1/2^	3.16	12.30	6.30	16.00
*δ_h_* (MPa)^1/2^	6.87	7.20	6.10	42.40
*δ* (MPa)^1/2^	19.48	22.96	20.20	47.90
*∆δ_j_*_−*p*_	–	9.15	3.24	37.86
*R_a_*	12.10	9.14	3.27	38. 09
*RED*	–	0.76	0.27	3.15
*χ_2_*_3_	–	1.79	0.23	31.00

**Table 2:** Physical properties of PC-DCM and PC-NMP dope solutions.

Polymer Concentration wt %	PC-NMP			Polymer Concentration wt %	PC-DCM		
	Density g/cm^3^	Viscosity mPa·s	Refractive Index		Density g/cm^3^	Viscosity mPa·s	Refractive Index
3	1.043	5.821	1.474	3	1.315	2.431	1.428
6	1.045	14.509	1.477	6	1.313	7.071	1.435
9	1.056	34.358	1.481	9	1.312	19.283	1.442
12	1.062	76.376	1.485	12	1.311	52.170	1.450
15	1.068	191.223	1.489	15	1.308	132.822	1.454
17	1.071	384.214	1.492	17	1.309	232.856	1.459
18	1.078	617.930	1.498	19	1.304	404.968	1.466
–	–	–	–	21	1.306	665.786	1.469

**Table 3:** Thermal properties of 18 wt % PC membranes prepared with different solvents.

S. No.	Membrane Name	Degradation Temperature °C		Residue %
		T_onset_	T_max_	
1	PC/NMP membrane	430.26	495.12	16.91
2	PC/DCM membrane	448.26	540.5	17.2

## Abbreviations

PC	Bisphenol A Polycarbonate
DCM	Dichloromethane
NMP	*N*-Methyl-2-Pyrrolidone
*RED*	Relative Energy Difference
FESEM	Field Emission Scanning Electron Microscope
FTIR	Fourier Transform Infrared Spectroscopy
TGA	Thermogravimetric analyzer (or analysis)

## Greek Symbols

*v* [cm^3^·mol^−1^]	The molar volume of ternary system component
*δ_d_* [(MPa)^1/2^]	The dispersive component of solubility parameter
*δ_h_* [(MPa)^1/2^]	The contribution due to hydrogen bonding
*δ_p_* [(MPa)^1/2^]	Polar component of solubility parameter
*δ* [(MPa)^1/2^]	Total solubility parameter
*χ*_13_	Nonsolvent/polymer interaction parameter
*χ*_23_	Solvent/polymer interaction parameter
*∆δ_i_*_–*p*_	Solubility parameter difference
*α*	Ideal selectivity
*R_a_*	Solubility parameter distance
*R_o_*	Interaction radius of polymer solubility sphere
*R*	Universal gas constant
*T*	Absolute Temperature
